# Anti-inflammatory activity of psoralen in human periodontal ligament cells via estrogen receptor signaling pathway

**DOI:** 10.1038/s41598-021-85145-1

**Published:** 2021-04-22

**Authors:** Huxiao Li, Jianrong Xu, Xiaotian Li, Yi Hu, Yue Liao, Wei Zhou, Zhongchen Song

**Affiliations:** 1 Department of Periodontology, Shanghai Ninth People’s Hospital, Shanghai Jiao Tong University School of Medicine, Shanghai, 200011 China; 2 Academy of Integrative Medicine, Shanghai University of Traditional Chinese Medicine, Shanghai, 201203 China; 3 Laboratory of Oral Microbiota and Systemic Diseases, Shanghai Research Institute of Stomatology,Shanghai Ninth People’s Hospital, Shanghai Jiao Tong University School of Medicine, Shanghai, 200011 China; 4 College of Stomatology, Shanghai Jiao Tong University; National Center for Stomatology; National Clinical Research Center for Oral Diseases; Shanghai Key Laboratory of Stomatology; Research Unit of Oral and Maxillofacial Regenerative Medicine, Chinese Academy of Medical Sciences, Shanghai, 200011 China; 5 Department of Pharmacology and Chemical Biology, Shanghai Jiao Tong University School of Medicine, Shanghai, 200025 China

**Keywords:** Cell biology, Molecular biology

## Abstract

Psoralen is one of the most effective ingredients extracted from the Chinese herb, *Psoralea corylifolia L*. Studies have found that psoralen has anti-inflammatory and estrogen-like effects; however, little research has been conducted to elucidate the mechanisms underlying these effects. Through the molecule docking assay, psoralen was found to have a better combination with ERα than ERβ. In human periodontal ligament cells, psoralen was found to upregulate the estrogen target genes (e.g., *CTSD, PGR, TFF1*) and down-regulate the expression of inflammatory cytokines (TNF-α, IL-1β, IL-6 and IL-8) stimulated by *P. gingivalis* LPS, as well as TLR4-IRAK4-NF-κb signaling pathway proteins. These effects were reversed by the ER antagonist ICI 182780. These results indicated that psoralen may exert anti-inflammatory effects as an agonist to ER, which could provide a theoretical basis for the use of psoralen for adjuvant therapy and prevention of periodontitis.

## Introduction

A large population of bacteria resides in the periodontal tissue; dental plaque and bacterial products can cause inflammation which results in periodontitis^1^. Periodontal disease may manifest as bleeding gums and tooth loss^2^. At present, scaling and root planing (SRP), the most basic treatment principle for periodontitis, is used to remove plaque and calculus. However, the application of SRP alone is unable to achieve satisfactory outcome and drug therapy could be a crucial supplement to SRP in the following situations^3^. Firstly, while treating patients with severe periodontitis, instruments cannot reach the deeper infection sites such as narrow and deep periodontal pockets, and the inflammation and alveolar bone resorption are also out of control. The application of drug therapy can help control infections in these deep periodontal pockets. Secondly, since the virulence factors can damage the epithelium of periodontal pockets, bacteria can easily invade the tissues and aggravate the absorption in alveolar bone. Mechanical therapy is difficult to completely remove infection in soft tissues, while topical drug therapy may have better effects. Thirdly, the acute inflammation of periodontal tissues needs to be relieved as soon as possible through drug therapy. Lastly, patients with systemic diseases need to control infection and prevent complications^4^. Systemic drug therapy can prevent the potential risk of infections^5^. Currently, various kinds of systemic antibiotics are widely used for periodontal drug therapy^6^. Some systemic antibiotics can strengthen the immune system by suppressing the target microbial species, but prolonged application of antibiotics could lead to obvious side effects such as antimicrobial resistance, imbalance of oral flora, and intestinal irritation^7–9^. Replacing the systemic antibiotics with drugs causing fewer side effects will be more promising.

**Figure 1:**
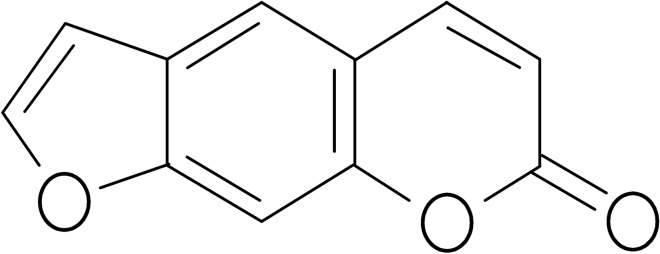
Molecular structure of fructus psoraleae’s extract psoralen.

*Psoralea corylifolia* L. is an annual herb distributed in the Yunnan and Sichuan provinces in China. An extensive amount of research has been conducted to evaluate the efficacy of Psoraleae (a fruit of *P. corylifolia*). It has been found that Psoraleae exhibits anti-tumor and anti-osteoporosis effects^10^. Psoralen , one of the main active constituents of Fructus Psoraleae (Fig. 1), was reported to have significant antibacterial effects on the periodontal pathogen *P. gingivalis,* and was found to inhibit the inflammatory response induced by it^11^. Psoraleae has also been shown to possess strong anti-osteoporosis effects^12^, inhibit tumor growth^13^, and exhibit estrogen-like effects^14^.

Estrogen is a sex hormone closely related to periodontitis. Alveolar bone density can be influenced by plasmatic estrogen levels, and it has been shown that lower estrogen levels were related to a more severe periodontal disease^15^. For women, decrease of estrogen levels after menopause can lead to postmenopausal osteoporosis^16^. During the treatment of periodontal disease, severer resorption of alveolar bone is observed in those osteoporotic patients, which increases the risk of tooth loss^17^. It has been reported that women receiving MHT (menopausal hormone therapy) have the lower risk of tooth loss^18^. After hormone therapy, postmenopausal women with chronic periodontitis were observed to have lower expression of inflammatory mediators IL-6 and IL1β in the gingival crevicular fluid, and better periodontal status^19^. An in vitro study has shown that, by treating periodontal ligament cells stimulated by LPS, estrogen can down-regulate the expression of TNF-α, IL-6, IL1β, and RANKL^20^. Therefore, during the development of periodontitis, estrogen plays a pivotal role in preventing the resorption of alveolar bone and modulating inflammatory mediators.

Despite that estrogen replacement therapy (ERT) in menopausal women can make a difference in controlling periodontitis, it cannot be applied to every periodontitis patient who needs drug therapy. Estrogen replacement therapy has the risk of increasing the incidence of uterine cancer and breast cancer^21^. Phytoestrogens, a group of plant derived compounds with estrogenic effect, have the potential to inhibit breast cancer^22^. As a phytoestrogen, psoralen can exert osteogenesis-potentiating effect and anti-inflammatory effect and avoid the risk of developing breast cancer^11–14^, which has a bright prospect of periodontal drug therapy. To the best of our knowledge, for psoralen, an extract of the Chinese herbal medicine Fructus Psoraleae, the mechanisms underlying its estrogen-like and anti-inflammatory effects have not been reported yet. The current study aimed to investigate the binding site of psoralen and the estrogen receptors to evaluate its effects on human periodontal ligament cells.

## Results

### The binding of psoralen to estrogen receptors

**Figure 2:**
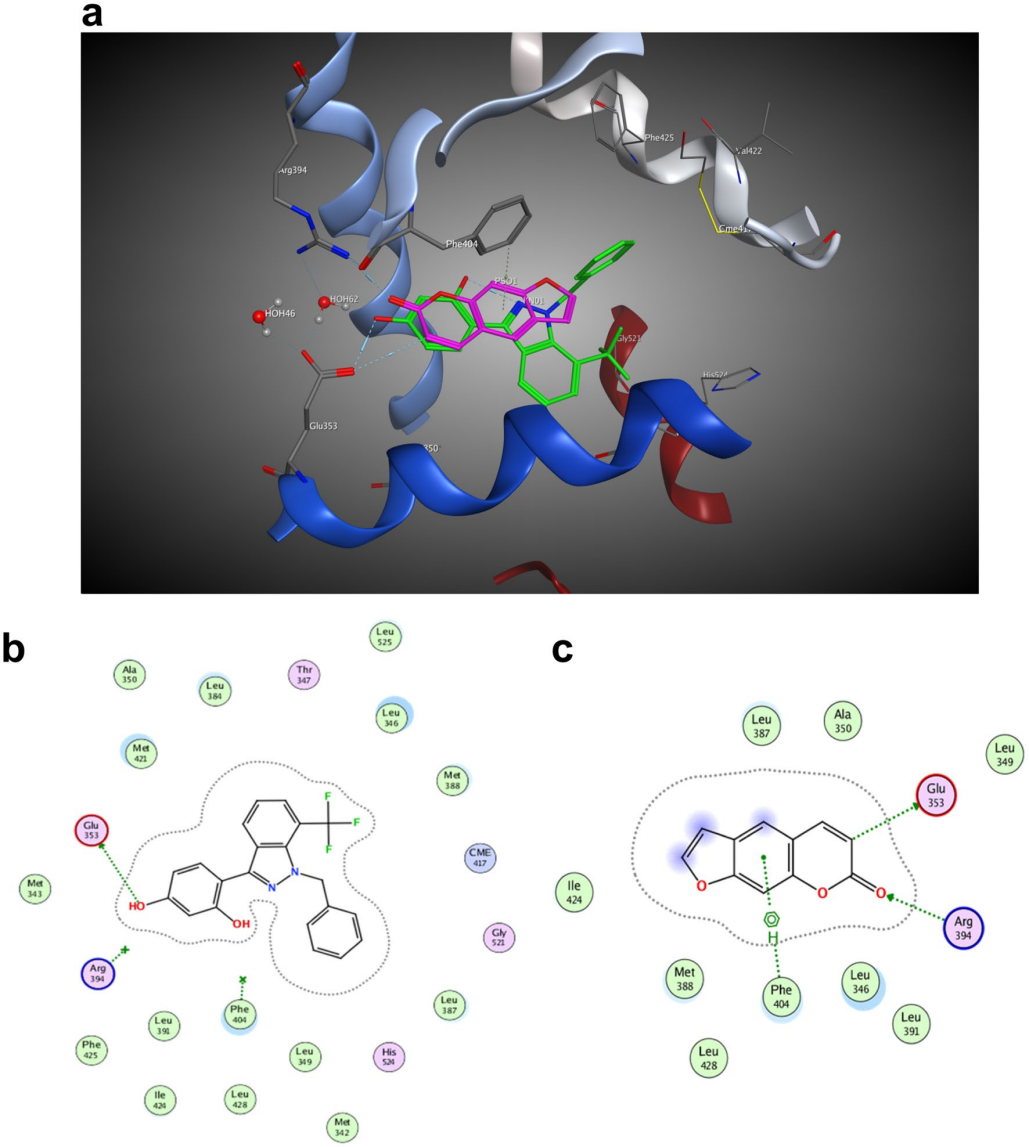
Psoralen and estradiol docked into ERα. (**a**) 3D image of psoralen and estradiol binding to the crystal structure of ERα; (**b**) estradiol binding to Glu 353, Arg 394 and Phe 404 of ERα; (**c**) Psoralen binding to Glu 353, Arg 394 and Phe 404 of ERα.

The 3D interaction diagram of the binding is shown in Fig. 2a. The binding mode of estradiol with ER-α is shown in Fig. 2b. Estradiol interacts with Glu353 by forming hydrogen bonds through the phenolic hydroxyl group. In this experiment, psoralen was docked into the pocket bound to the ER-α ligand. By analyzing their binding mode, we found that the binding of psoralen to ERα was similar to estradiol. Estradiol and psoralen could dock into ERα via the binding sites Glu353, Arg394, and Phe404 (Fig. 2c). As shown in Fig. 2c, there are hydrogen bonds between the hydrogen atom on the psoralen ester α carbon and Glu353. The carbonyl group of the ester bond and Arg394 formed hydrogen bonding interactions. The carbonyl group of Glu353 and psoralen acted as a hydrogen bond receptor; the aromatic ring structure of psoralen and Phe404 had a stacking interaction of CH-π. The results showed that psoralen could match the binding pocket of ER-α, which was similar to estradiol.

**Figure 3:**
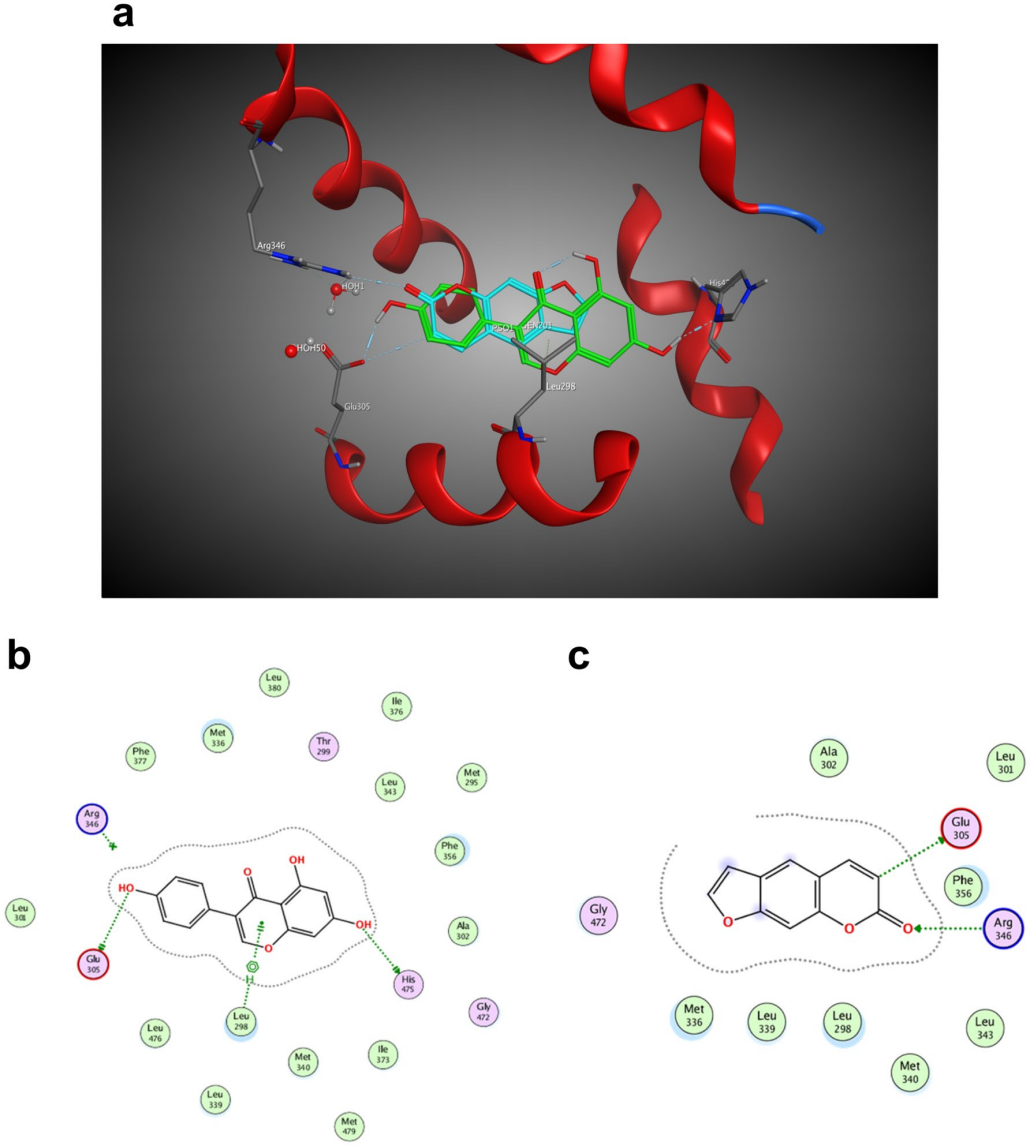
Estradiol and Psoralen docked with ERβ. (**a**) 3D image of psoralen and estrogen binding to the crystal structure of ERβ; (**b**) estradiol binding to Glu305, Arg346, Leu298 and His475 of ERβ; (**c**) Psoralen binding to Glu305 and Arg346 of ERβ.

The 3D interaction diagram of the binding is shown in Fig. 3a. Glu305, Arg346, Leu298 and His475 were the hot residues that were involved in estradiol binding with ERβ (Fig. 3b, c). It was found that psoralen docked poorly with ERβ through binding with Glu305 and Arg346. The results suggested that psoralen did not bind well with ERβ.

### Identification of hPDLCs

**Figure 4:**
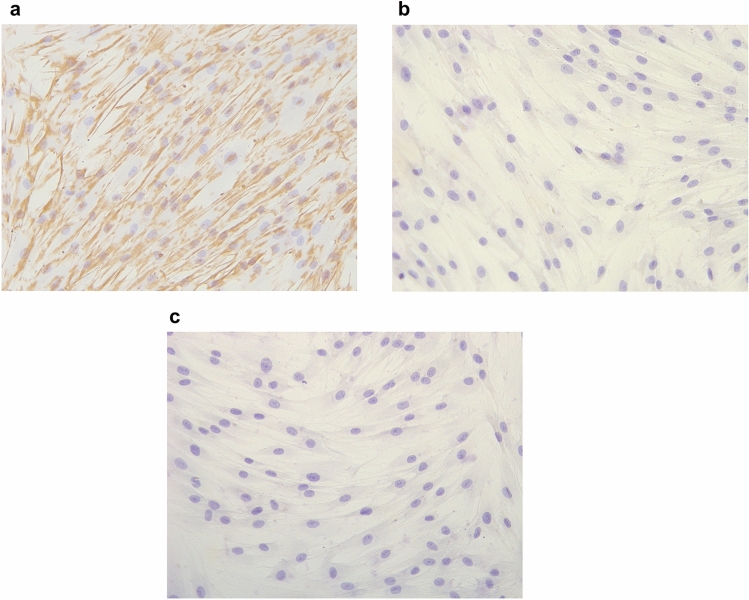
Immunohistochemical stainings for identifying hPDLCs (×200). The positive expression of vimentin was observed in cells, presenting as brownish yellow cytoplasm (**a**). Negetive control group was shown in (**b**). The cells incubated with pan cytokerain primary antibodies failed to stain (**c**).

The third-generation cells cultured in vitro were stained through SP immunohistochemical staining technique. The results showed that the positive expression of vimentin was observed in cells, presenting as brownish yellow cytoplasm (Fig. 4a). Negative control group was shown in Fig. 4b. The cells incubated with pan cytokeratin primary antibodies failed to stain (Fig. 4c). It meant that cells were stained positive for vimentin and negative for pan cytokeratin. The results suggested that the obtained cells were derived from mesoderm mesenchyme^23^, which proved that the isolated cells were indeed derived from the periodontal ligament.

### Effects of psoralen on viability of hPDLCs

**Figure 5:**
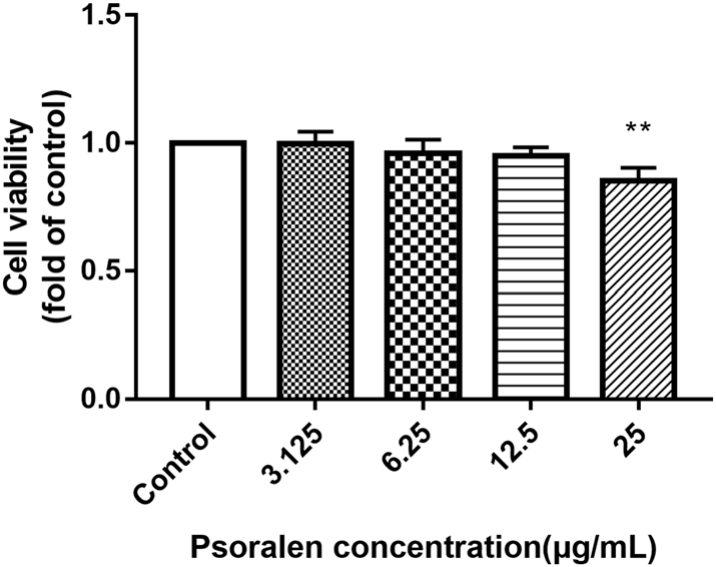
Effects of psoralen on the cell viability of hPDLCs. (**p < 0.01 compared with the control group).

The MTT assay was performed to detect the cell viability after treatment with psoralen. At a concentration of 25 µg/mL, psoralen had a negative impact on the survival of cells as shown in Fig. 5. Psoralen at a concentration of 12.5 μg/mL or less, did not have a significant effect on the cell viability of hPDLCs.

### Psoralen enhanced estrogen target genes expression of hPDLCs

**Figure 6:**
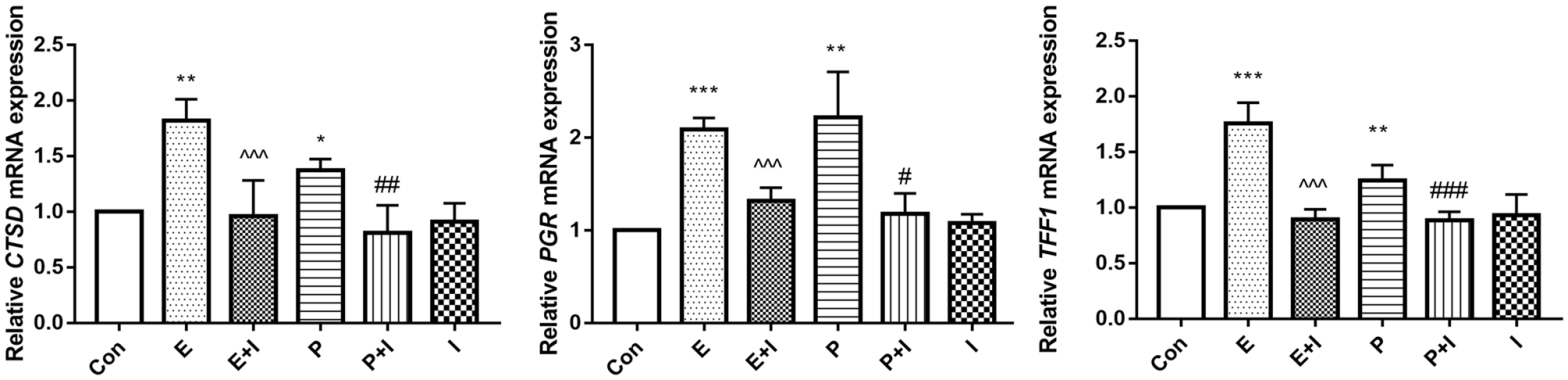
Effects of psoralen on the expression of estrogen target genes in hPDLCs. Cells were divided into 6 groups: Con, control group; E, estrogen (10^–8^ M) group; P, psoralen (12.5 μg/mL) group; E + I, estrogen (10^–8^ M) + I (1 μM) group; P + I, psoralen (12.5 μg/mL) + I (1 μM) group; I, I (1 μM) group (*p < 0.05,**p < 0.01,***p < 0.001 compared with the control group, ^#^*p* < 0.05, ^##^*p* < 0.01, ^###^*p* < 0.001 compared with the P group, ^^^*p* < 0.05, ^^^^*p* < 0.01, ^^^^^*p* < 0.001 compared with the E group).

As mentioned above, psoralen docks with ERα better than it docks to ERβ. Estrogen target genes Cathepsin D (*CTSD*), progesterone receptor (*PGR*), and trefoil factor 1(*TFF1*) were selected to verify the molecular docking results. As shown in Fig. 6, estradiol could significantly upregulate the expression of the ERα target genes (*CTSD*, *PGR*, and *TFF1*); however, when the cells were treated with the ER antagonist,the effect of estrogen on activating target genes would be blocked (*p* < 0.01). Similar with estradiol, psoralen could also upregulate the expression of estrogen target genes , which were blocked by ER antaonist (*p* < 0.01). The results indicated that psoralen had estrogen-like effects.

### Psoralen inhibited mRNA expression of inflammatory cytokines in hPDLCs

**Figure 7:**
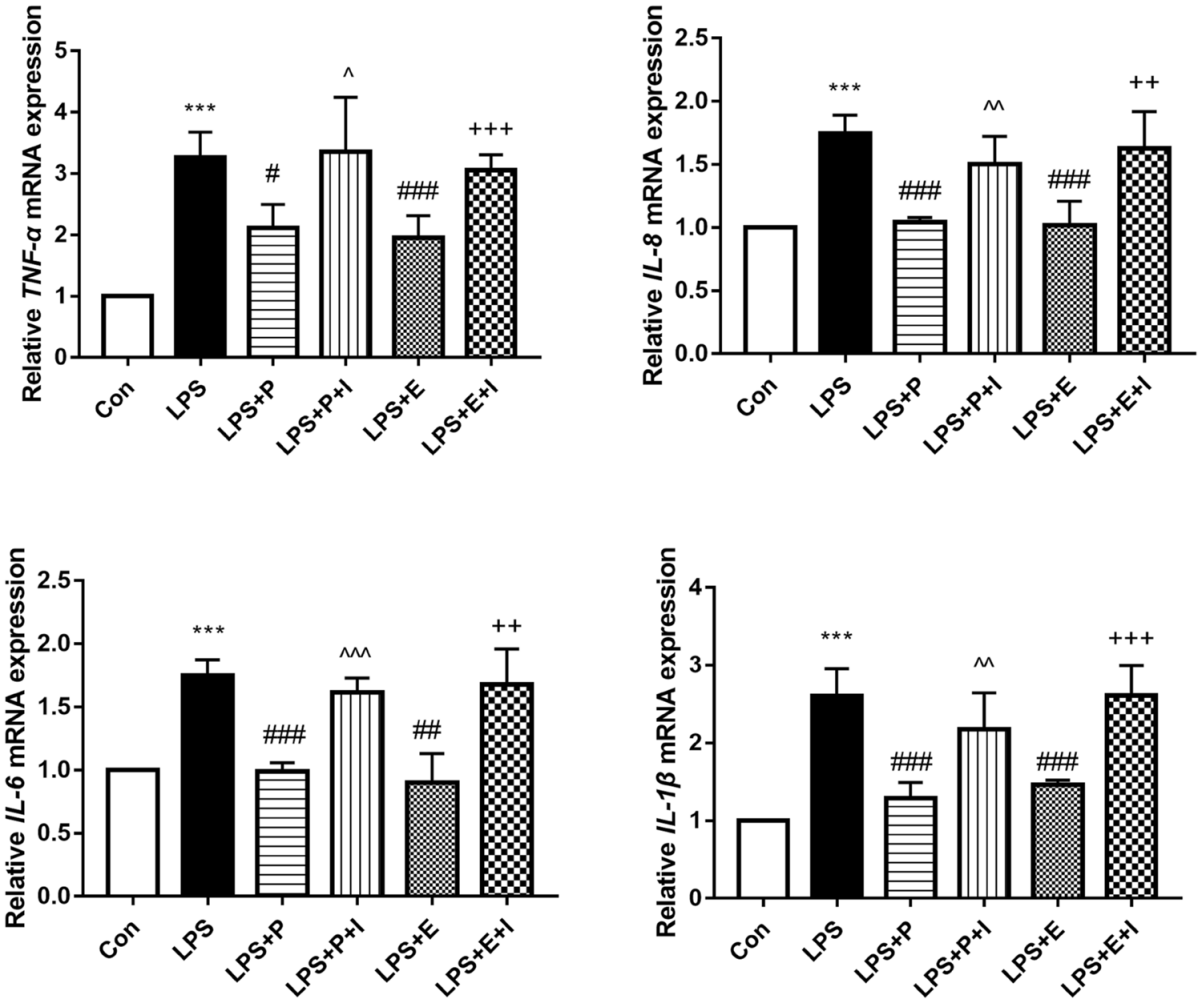
Effects of psoralen on the expression of inflammatory cytokines mRNA. hPDLCs were divided into 6 groups: Con, control group; LPS, LPS (1 μg/mL) group; LPS + E, LPS and estrogen (10^–8^ M) group; LPS + P, LPS and psoralen (12.5 μg/mL) group; LPS + E + I, LPS and estrogen (10^–8^ M) and ICI (1 μM) group; LPS + P + I, LPS and psoralen (12.5 μg/mL) and ICI (1 μM) group (*p < 0.05,**p < 0.01, ***p < 0.001 compared with the control group, ^#^*p* < 0.05, ^##^*p* < 0.01, ^###^*p* < 0.001 compared with the LPS group, ^^^*p* < 0.05, ^^^^*p* < 0.01, ^^^^^*p* < 0.001 compared with the LPS + P group, ^+^*p* < 0.05, ^++^*p* < 0.01, ^+++^*p* < 0.001 compared with the LPS + E group).

Compared with the control group, *P. gingivalis* LPS could significantly increase the mRNA expression of inflammatory cytokines (*TNF-α*, *IL-1β*, *IL-8* and *IL-6*) in hPDLCs (*p* < 0.001). The expression of these mRNA in hPDLCs could significantly be inhibited by psoralen or estradiol. Meanwhile, the anti-inflammatory effects of psoralen and estradiol could be partially reversed by the ER antagonist, which increased the expression of inflammatory cytokines (Fig. 7). These data indicated that psoralen had anti-inflammatory effects, which could be partially reversed by ER antagonist.

### Psoralen inhibited protein levels of inflammatory cytokines released by hPDLCs

**Figure 8:**
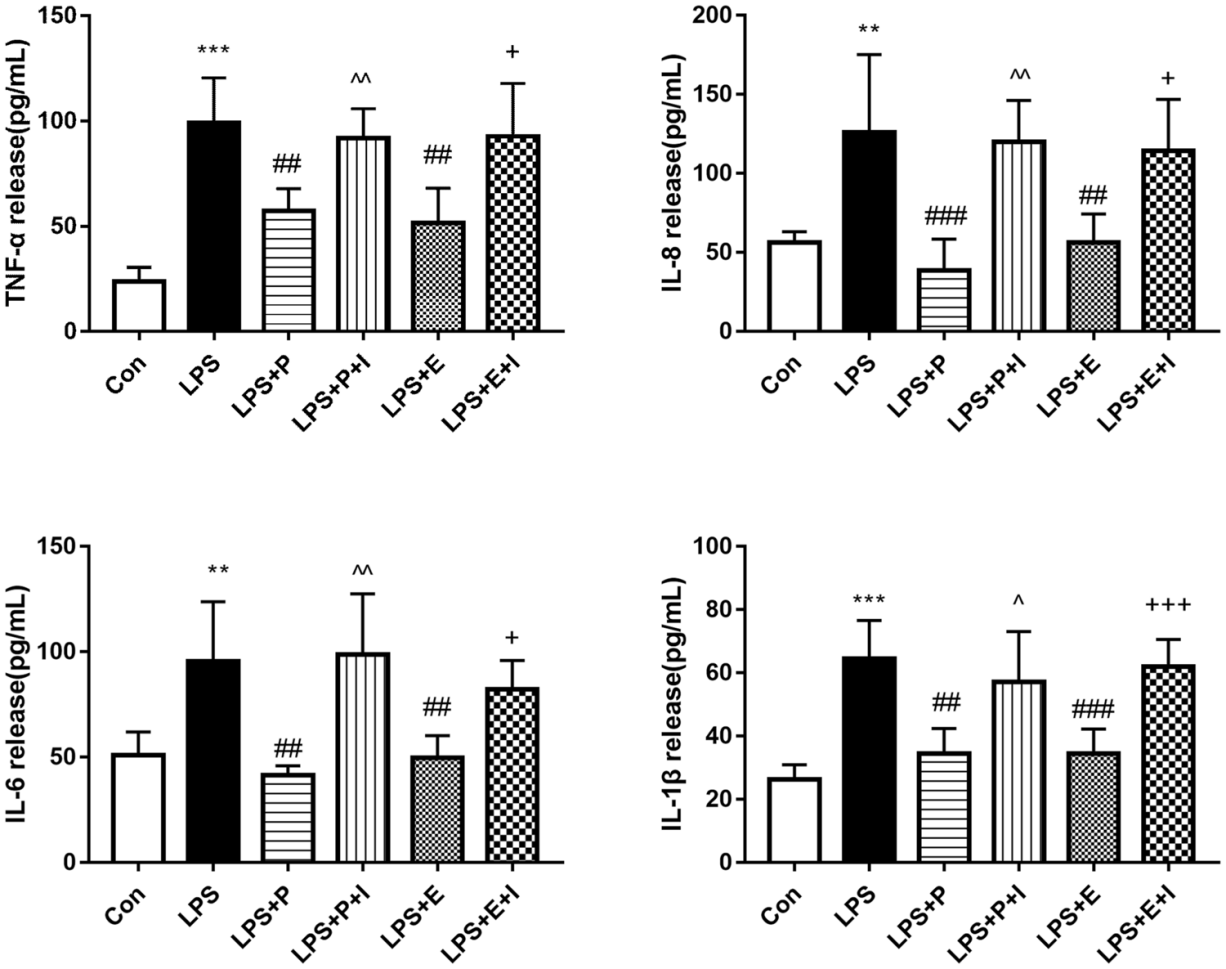
Effects of psoralen on the expression of inflammatory factors protein. hPDLCs were divided into 6 groups: Con, control group; LPS, LPS (1 μg/mL) group; LPS + E, LPS and estrogen (10^–8^ M) group; LPS + P, LPS and psoralen (12.5 μg/mL) group; LPS + E + I, LPS and estrogen (10^–8^ M) and ICI (1 μM) group; LPS + P + I, LPS and psoralen (12.5 μg/mL) and ICI (1 μM) group (*p < 0.05,**p < 0.01, ***p < 0.001 compared with the control group, ^#^*p* < 0.05 compared with the LPS group, ^^^*p* < 0.05, ^^^^*p* < 0.01, compared with the LPS + P group, ^+^*p* < 0.05, ^++^*p* < 0.01 compared with the LPS + E group).

Compared with the control group, the protein levels of inflammatory cytokines (TNF-α, IL-1β, IL-8, and IL-6) released by hPDLCs increased significantly with the stimulation of *P. gingivalis* LPS. These inflammatory cytokines protein levels were downregulated by psoralen or estradiol. The ER antagonist could reverse the decline, indicating it could block the anti-inflammatory effects of psoralen and estradiol (Fig. 8). The results showed that psoralen could reduce the protein levels of inflammatory cytokines (TNF-α, IL-1β, IL-8, and IL-6), which could be reversed by the ER antagonist.

### Psoralen inhibited the activation of TLR4/NF-κB signaling pathway

**Figure 9:**
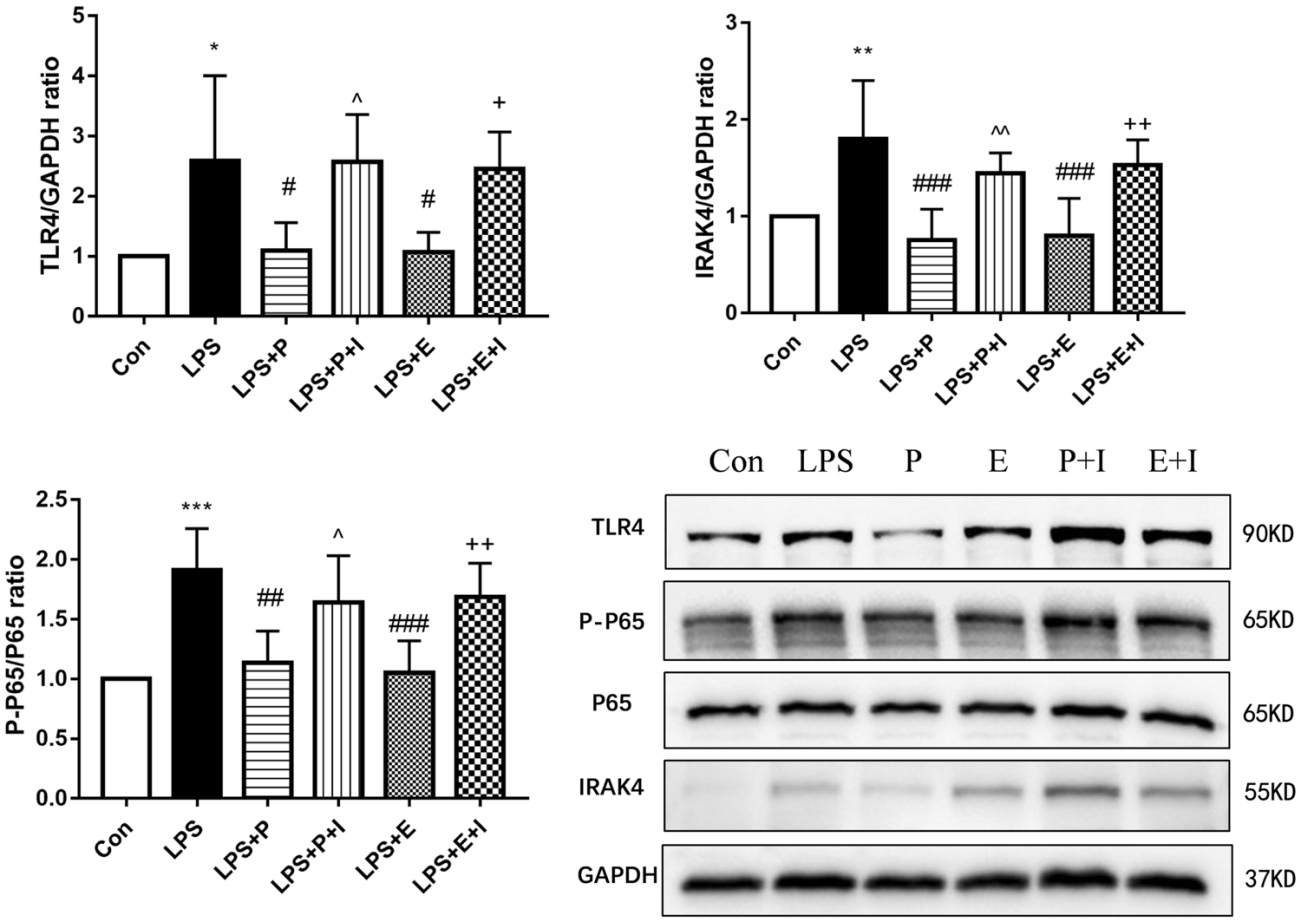
Effects of psoralen on the expression of TLR4, IRAK4, p65 and p-p65 protein. hPDLCs were divided into 6 groups: Con, control group; LPS, LPS (1 μg/mL) group; LPS + E, LPS and estrogen (10^−8^ M) group; LPS + P, LPS and psoralen (12.5 μg/mL) group; LPS + E + I, LPS and estrogen (10^−8^ M) and ICI (1 μM) group; LPS + P + I, LPS and psoralen (12.5 μg/mL) and ICI (1 μM) group (*p < 0.05,**p < 0.01, ***p < 0.001 compared with the control group, ^#^*p* < 0.05, ^##^*p* < 0.01, ^###^*p* < 0.001 compared with the LPS group, ^^^*p* < 0.05, ^^^^*p* < 0.01 compared with the LPS + P group, ^+^*p* < 0.05 compared with the LPS + E group).

The Fig. 9 showed that in hPDLCs, the protein levels of TLR4, IRAK4, p65, and p-p65 increased, which were stimulated by the *P. gingivalis*-LPS . However, the psoralen and estrogen groups showed lower protein levels (p < 0.01), which suggested that psoralen could inhibit the activation of NF‐κB signaling pathway. When pre-treated with the ER antagonists, the protein levels partially reversed. These results indicated that psoralen exhibited anti-inflammatory effects by inhibiting the TLR4/NF-κB signaling pathway, which might relate to its estrogen-like effects.

## Discussion

Periodontitis is a chronic infectious disease. Phytoestrogens are plant-based compounds with anti-inflammatory effects and potential for treating periodontitis. Our study found that psoralen could combine with ERα, which was better than ERβ, through molecule docking. The study was the first to investigate the estrogen-like and anti-inflammatory effects of psoralen on *P.gingivalis*-LPS induced periodontal inflammation. The results showed that psoralen antagonized the expression of inflammatory cytokines such as TNF-α and ILs secreted by hPDLCs induced by *P. gingivalis*-LPS at both mRNA and protein levels, and it could inhibit the TLR4/NF-κB signaling pathway.

Plaque biofilm is the initial factor for periodontitis^24^. *P. gingivalis* is one of the main periodontal pathogens of periodontitis. *P. gingivalis* LPS can stimulate immune cells such as monocytes and macrophages, produce proinflammatory cytokines, destroy periodontal collagen fibers, and cause destruction of periodontal tissues^25,26^. In addition to the direct consequences of bacterial infection, the immune response of the host is also a major cause of periodontal tissue injury^27^. Immune responses can be divided into innate immune responses and acquired immune responses^28^. As a pattern recognition receptor, TLR4 is part of the innate immune response that is involved in activating inflammatory responses. LPS can be recognized by TLR4 and can initiate a pathway causing the release of a series of inflammatory cytokines^29^. These inflammatory cytokines include IL-1, IL-6, IL-8, and TNF-α, etc. are involved in promoting chemotaxis and adhesion of leukocytes, activating mononuclear macrophages, and increasing the production of prostaglandins and matrix metalloproteinases, etc.^30^. Osteoclasts and matrix metalloproteinases that are stimulated by these inflammatory factors may cause damage to tissues. These injuries cannot be cured by mechanotherapy alone, which is why drug therapy plays an important role in healing them. Pathogenic bacteria exist not only in deep periodontal pockets, so systemic antibiotics may be used as complements to periodontal treatment. But long-term administration of antibiotics might have side effects like increasing antimicrobial resistance and imbalance of oral and intestinal flora. Replacement drugs with less side effects and anti-inflammatory effects will be more promising.

There are many Chinese medicine extracts that can serve as replacement drugs. Our previous research showed that psoralen had antibacterial and anti-inflammatory effects^11^. Wang et al.^31^ found that psoralen could relieve osteoarthritis. Zhou et al.^32^ also found that psoralen effectively inhibited the activation of NF-κB and down-regulated the expression of TGF-β1 in LO2 cells. Since estrogen also exerts strong anti-inflammatory effects^33^, we assumed that there was a link between psoralen’s anti-inflammatory and estrogen-like effects. It was reported that TNF level of human peripheral blood mononuclear cells upregulated by LPS could be inhibited by estradiol. The estrogen concentration ranges from 10^–10^ to 10^–7^ M in men and 10^–8^ to 10^–7^ M in women^34^; 10^–10^ to 10^–8^ M of estradiol could down-regulate the expression of IL-6, TNF, IL-1β, and the ratio of IL-1β/IL-1 receptor antagonist (IL-1RA)^35^. On the contrary, a lack of estradiol could lead to an increase in the secretion of TNF, IL-1β, and IL-6 in human monocytes (THP-1)^36^. In addition, the ER could exert anti-inflammatory effects by inhibiting the activity of NF-κB^37^. These studies indicate that estradiol could inhibit the production of inflammatory cytokines. However, as a sex hormone, estrogen promotes breast tumorigenesis, which is inappropriate to be used as an anti-inflammatory drug. But psoralen has the potential to become the drug that can inhibit inflammatory with anti-tumor effect^38^.

It was reported that psoralen could increase the expression of ERα in osteoclasts^39^. Estrogen receptors are divided into estrogen receptor α (ERα) and estrogen receptor β (ERβ). ERα is generally expressed in classical targeted tissues, such as breast tissue. ERα and ERβ have both been detected in hPDLCs^40^. The effects of psoralen might be related to the ER, but there was no direct evidence to confirm the interaction of psoralen and estrogen receptors. The molecule docking assay conducted by MOE provided evidence and showed valuable hints for further experiment; the result suggested us to focus on psoralen binding to ERα. MOE developed by the Chemical Computing Group is a software for molecular modelling and computer-assisted drug design. It is widely used in the fields of chemistry and biopharmaceuticals. It can simulate the binding of psoralen molecules and receptors to find the docking site.

In this experiment, the molecular structure of psoralen was simulated by the MOE software. The results suggested that psoralen and ERα bound well, and the combination was similar to that of estradiol and ERα. After estradiol docking with the ER, the complex would bind to the estrogen response element and regulate the expression of the target genes. Cathepsin D (*CTSD*), progesterone receptor (*PGR*), and trefoil factor 1 (*TFF1*) were target genes mediated by ERα^41^. In this study, the expressions of *CTSD*, *PGR*, and *TFF1* mRNA were significantly upregulated with the treatment of psoralen and estrogen. The results indicated that psoralen exerted its estrogen-like effects through docking with ERα. The addition of ER antagonists blocked the upregulation of psoralen on the target genes, which also supported the similarity between psoralen and estrogen. The results verified the molecular docking assay, suggesting that psoralen could exert estrogen-like effects through docking with ERα and provoked ERα to regulate the expression of target genes.

*P. gingivalis*-LPS could bind to TLR4 and initiate a cascade reaction, which leads to the upregulation of a series of inflammatory cytokines^42^. Toll-like receptors are key components of innate immunity, which can detect and respond to pathogens^43^. TLR4 signals recognize LPS via two different pathways. One of them is the MyD88-dependent pathway. IRAK1, IRAK4, and TRAF6 are recruited by MyD88. Phosphorylated IRAK1 and the ubiquitination of TRAF6 can activate NF-κB. The NF‐κB signaling pathway is the pivotal pathway that participates in inflammation and regulation of the host’s immune response. TLR2 also participate in the signaling pathway through MyD88^44^. The anti-inflammatory mechanisms of estrogen are complex and diverse. While the LPS of *P. gingivalis* is recognized by TLR4 to initiate a downstream immune inflammatory response^43^; estrogen can inhibit the production of inflammatory factors by blocking TLR4^45^. In addition to block Toll-like receptors, it can also inhibit NF-κB activity^46^. It indicates that psoralen with estrogen-like effects may also influence TLR4 and NF-κB. The results showed that the activation of TLR4/NF-κB signaling pathway induced by *P. gingivalis*-LPS could be inhibited by psoralen, which could also be blocked by ER antagonists. These findings collectively demonstrated that psoralen could exert estrogen-like effects and inhibit the TLR4/NF‐κB signaling pathway to down-regulate the levels of inflammatory cytokines in hPDLCs with inflammatory status.

## Conclusion

Psoralen could exert anti-inflammatory effects through the combination with ER. Its mechanism of anti-inflammatory effects was related to the inhibition of activation of the TLR4-IRAK4-NF-κB pathway. The results could provide a theoretical basis for the use of psoralen for adjuvan therapy and prevention of periodontitis. Psoralen may have the potential as a new anti-inflammatory drug for the treatment of periodontitis.

## Materials and methods

### Molecular docking

The binding mode of psoralen, estrogen, and estrogen receptors were analyzed by molecular modelling software, Molecular Operating Environment (MOE 2014.09, Canada)^47,48^. The structures of ERα (PDB: 3OS8) and ERβ (PDB: 1X7J) were retrieved from the RCSB protein database for molecular docking studies. Through the conformation search algorithm, 30 preponderant psoralen conformations were generated. We adopted the automated docking procedure in MOE and utilized default parameters. Psoralen was docked into the binding pockets of ERα and Erβ, respectively. The best ligand–target binding mode was generated by minimizing the energy and assessing the scores of the docking poses. The interaction diagrams of the binding modes were illustrated by using MOE.

### Culture of human periodontal ligament cells

Primary hPDLCs were obtained and cultured from the ligament tissues in the middle of the premolar roots using a tissue explant method^11^. All patients agreed to participate in the study and signed the informed consent forms. After tooth extraction, these teeth were washed at least five times with sterile PBS. Then the ligament tissues in the middle of the teeth root were scraped off and cut into pieces. The tissues were placed at the bottom of a Petri dish. Four hours later, 4 mL Dulbecco’s Modified Eagle’s medium (DMEM) (Hyclone, USA) with 10% fetal bovine serum (FBS) (Gibco, USA) and 100 μg/mL streptomycin (Beyotime Biotechnology, China) was added. Cells between 3 and 5 passage were used for the following experiments.

### Immunohistochemical stainings

The third-generation human periodontal ligament cells were seeded in 24-well microplates at a density of 8 × 10^4^ cells per well. The cells were fixed in 4% paraformaldehyde for 15–30 min and immunohistochemical stained according to the operating instructions of the UltraSensitive SP IHC Kit (Maixin biotechnology company, Fuzhou, China). Fixed cells were blocked with 10% goat serum incubated with primary antibodies (vimentin and pan cytokeratin) for 60 min at room temperature. After rinsed by PBS, cells were incubated with corresponding secondary antibodies for 10 min at room temperature. Then cells were counterstained with 3,3′-diaminobenzidine (DAB) and hematoxylin. Images were taken under a microscope with Leica camera.

### Cell viability assay

The MTT assay was conducted to evaluate whether psoralen (Aladdin Chemical Company, Shanghai, China) was toxic on hPDLCs. Psoralen was dissolved in dimethyl sulfoxide (DMSO) (Sigma, USA). hPDLCs were collected by trypsinization and seeded into a 96-well microplate at a density of 10^4^ per well. After 24 h incubation, the cells were treated for another 24 h with psoralen at different concentrations of 3.125 μg/mL, 6.25 μg/mL, 12.5 μg/mL and 25 μg/mL. Then 200 µl MTT (0.5 mg/mL) dilution was added to each well and the microplate was put in the incubator in the dark for 4 h. An equal volume of DMSO was added after extracting the MTT liquid, then the microplate was shaken for 10 min to dissolve the crystals. The absorbance at 490 nm was observed with microplate reader (Bio-Tek, USA).

### RNA extraction and RT-PCR analysis

hPDLCs were seeded in 6-well microplates at a density of 4 × 10^5^ per well. The RNA was extracted from hPDLCs using the Total RNA Kit (Omega Bio-Tek, Inc., Norcross, GA, USA) according to the manufacturer’s instructions. The purity and concentration were measured by a spectrophotometer (NanoDrop ND-1000; NanoDrop Technologies, Wilmington, DE, USA). The total RNA (1000 ng) was used in reverse transcription. Subsequently, an RT-PCR assay was performed using SYBR Premix Ex Taq (Takara, Kusatsu, Shiga, Japan) on a Roche LightCycler 480 Real-Time PCR Detection System (Roche, Basel, Switzerland) according to the manufacturer’s protocol. The sequences of genes including *TNF-α*, *IL-1β*, *IL-6*, *IL-8*, *PGR*, *CTSD*, *TFF1* and their primer pairs were listed in Table 1.

**Table1:** Primers of target genes.

Target genes	Primers (all from 5′–3′)
*TNF-α*	Forward: TTCTGTCTACTGAACTTCGGGGTGATCGGT
	Reverse: GTATGAGATAGCAAATCGGATGACGGTGTG
*IL-1β*	Forward: TGATGGCTTATTACAGTGGC
	Reverse: GTAGTGGTGGTCGGAGATT
*IL-6*	Forward: CTAGAGTACCTCCAGAACAGATTTGA
	Reverse: TCAGCAGGCTGGCATTT
*IL-8*	Forward: ACTCCAAACCTTTCCACC
	Reverse: CTTCTCCACAACCCTCTG
*PGR*	Forward: AGCCAGAGCCCACAATACAG
	Reverse: CCCACAGGTAAGGACACCAT
*CTSD*	Forward: TGACCGTGACAACAACAGG
	Reverse: TGCTCTGGGACTCTCCTCTG
*TFF1*	Forward: TCCCCTGGTGCTTCTATCCT
	Reverse: GGACTAATCACCGTGCTGGG
*GAPDH*	Forward: CGGGAAACTGTGGCGTGAT
	Reverse: GTCGCTGTTGAAGTCAGAGGAG

### Enzyme linked immunosorbent assay (ELISA)

For the enzyme‐linked immunoassay, hPDLCs were seeded in 6-well microplates at a density of 4 × 10^5^ per well. hPDLCs were pre-treated with E2, ICI or psoralen (12.5 μg/mL) for 2 h and then stimulated by 1 μg/mL *P. gingivalis*-LPS for 24 h. The supernatant medium was collected for measurement. After centrifuging, the release of TNF-α, IL-1β, IL-6 and IL-8 in the supernatant medium was measured by ELISA kits (UBI, Sunnyvale, CA, USA) according to the manufacturer’s instructions.

### Western blot

Western blotting analysis was applied to detect the expression of TLR-4, IRAK4, p65 and pp65. Cells were plated on a 6 cm culture dish at a density of 8 × 10^5^ cells per dish. The hPDLCs were collected and lysed in cold RIPA containing 1% protease inhibitor cocktail and 1% PMSF (Beyotime, Shanghai, China). The protein concentration was detected via a BCA protein assay kit (Beyotime, China). Then, equal amounts of each protein sample were separated via 10% sodium dodecyl sulfate‐polyacrylamide gel electrophoresis and transferred onto PVDF membrane blocked with 5% skimmed milk as previously described^9^. Proteins were probed with appropriate antibodies including anti-TLR4 (1:1000, abs132000; Cell Signaling Technology, USA), anti-IRAK4 (1:1000, abs143411; Cell Signaling Technology, USA), anti-p65 (1:1000, no. 8242; Cell Signaling Technology, USA), anti-p-p65 (1:1000, no. 8242; Cell Signaling Technology, USA) and anti-GAPDH (1:1000, AB-P-R001, Cell Signaling Technology, USA). The data were quantified using the Image Studio Lite ver. 5.2 software (Supplementary Information 1).

### Statistical analysis

All experiments were repeated at least three times. All values are presented as mean ± SD. The statistical analysis involved one-way analysis of variance (ANOVA), Kruskal–Wallis test and Mann–Whitney test. A *p* value < 0.05 was considered statistically significant.

### Ethics approval

The studies involving human participants were reviewed and approved by Shanghai Ninth People’s Hospital, and all methods were performed in accordance with the appropriate guidelines and regulations. The Institute Review board number is 2018-120-T98. The patients/participants provided their written informed consent to participate in this study.

## Supplementary Information

Supplementary Information

## Data Availability

The datasets generated during and/or analysed during the current study are available from the corresponding author on reasonable request.
