# Supplementary material for: Anti-inflammatory activity of psoralen in human periodontal ligament cells via estrogen receptor signaling pathway
Source: Sci Rep. 2021 Apr 22;11:8754. doi: 10.1038/s41598-021-85145-1 (PMC8062431; doi:10.1038/s41598-021-85145-1)

Anti-inflammatory activity of psoralen in human periodontal ligament cells via estrogen receptor signaling pathway

Author list：

1. Huxiao Li#
tentigerli@163.com
Department of Periodontology, Shanghai Ninth People's Hospital, College of Stomatology, Shanghai JiaoTong University School of Medicine, Shanghai 200001, China
Shanghai Key Laboratory of Stomatology & Shanghai Research Institute of Stomatology, National Clinical Research Center for Oral Diseases, Shanghai 200001, China

2. Jianrong Xu#
janker.xu@gmail.com
Academy of Integrative Medicine, Shanghai University of Traditional Chinese Medicine, Shanghai 201203, China.
Department of Pharmacology and Chemical Biology, Shanghai Jiao Tong University School of Medicine, Shanghai 200025, China

3. Xiaotian Li
1012452270@qq.com
Department of Periodontology, Shanghai Ninth People's Hospital, College of Stomatology, Shanghai JiaoTong University School of Medicine, Shanghai 200001, China
Shanghai Key Laboratory of Stomatology & Shanghai Research Institute of Stomatology, National Clinical Research Center for Oral Diseases, Shanghai 200001, China

4. Yi Hu
hy090508@163.com
Department of Periodontology, Shanghai Ninth People's Hospital, College of Stomatology, Shanghai JiaoTong University School of Medicine, Shanghai 200001, China
Shanghai Key Laboratory of Stomatology & Shanghai Research Institute of Stomatology, National Clinical Research Center for Oral Diseases, Shanghai 200001, China

5. Yue Liao
vickyliao0911@163.com
Department of Periodontology, Shanghai Ninth People's Hospital, College of Stomatology, Shanghai JiaoTong University School of Medicine, Shanghai 200001, China

Shanghai Key Laboratory of Stomatology & Shanghai Research Institute of Stomatology, National Clinical Research Center for Oral Diseases, Shanghai 200001, China

6. Wei Zhou*
sweetzw@hotmail.com
Laboratory of Oral Microbiota and Systemic Diseases, Shanghai Research Institute of Stomatology, Ninth People's Hospital, Shanghai JiaoTong University School of Medicine, Shanghai 200120, China
Shanghai Key Laboratory of Stomatology & Shanghai Research Institute of Stomatology, National Clinical Research Center for Oral Diseases, Shanghai 200001, China

7. Zhongchen Song*
szhongchen@sina.com
Department of Periodontology, Shanghai Ninth People's Hospital, College of Stomatology, Shanghai JiaoTong University School of Medicine, Shanghai 200001, China
Shanghai Key Laboratory of Stomatology & Shanghai Research Institute of Stomatology, National Clinical Research Center for Oral Diseases, Shanghai 200001, China

# Huxiao Li and Jianrong Xu have contributed equally to this work.

* Co-coresponding author: Wei Zhou: sweetzw@hotmail.com; Zhongchen Song: szhongchen@sina.com

GAPDH For TLR4

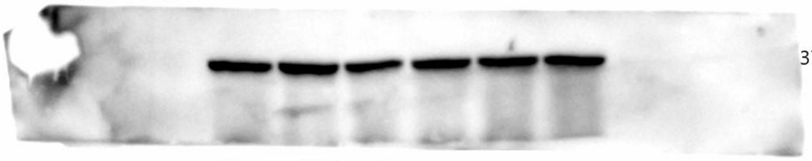

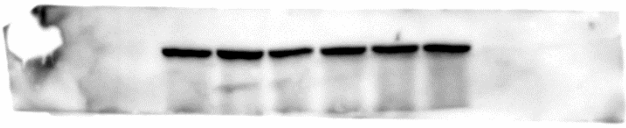

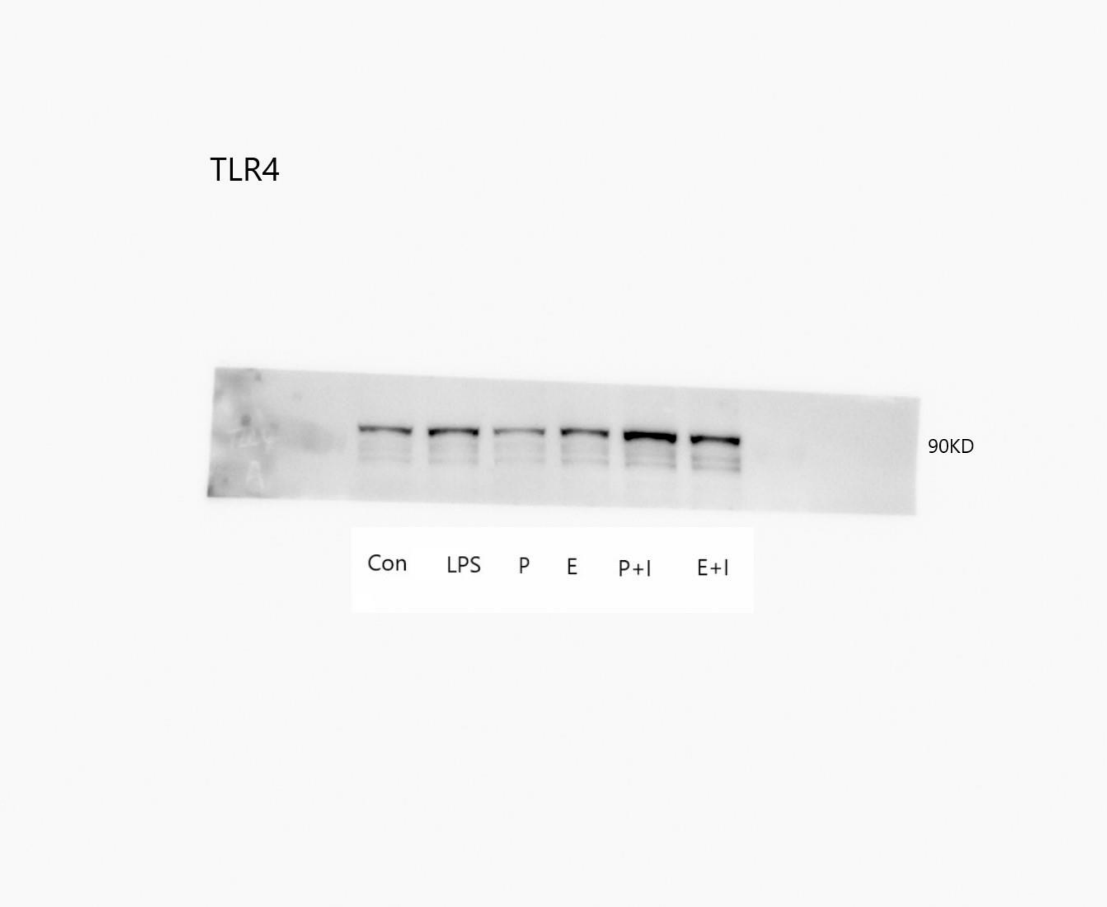
TLR4
90KD
Con
LPS
P
E
P+I
E+I

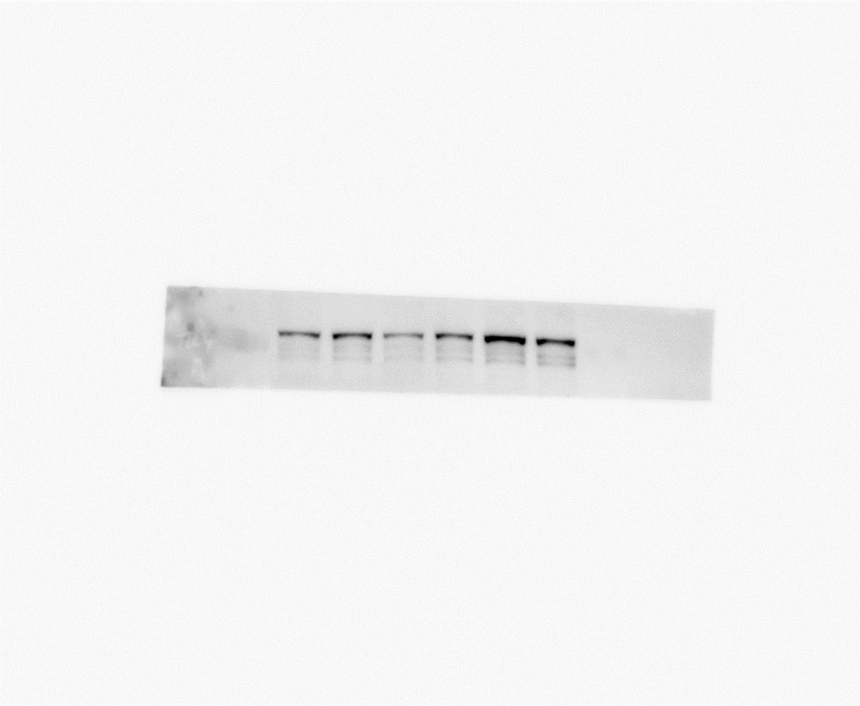

GAPDH For P65

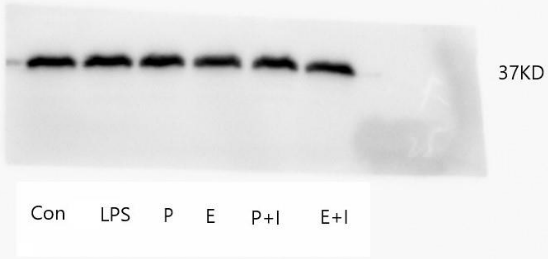

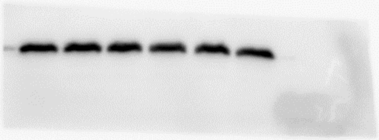

P65

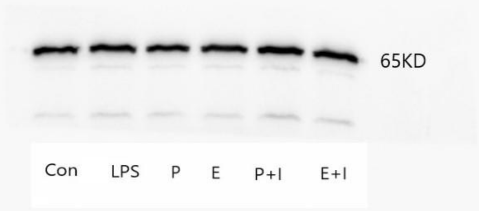

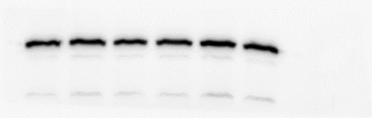

GAPDH For

P-P65

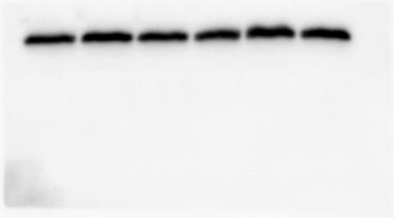

Con LPS P E P+I E+I

GAPDH For

IRAK4

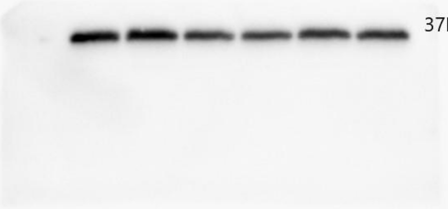

Con LPS P E P+I E+I

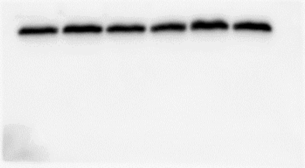

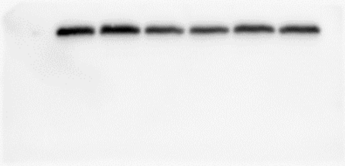

IRAK4

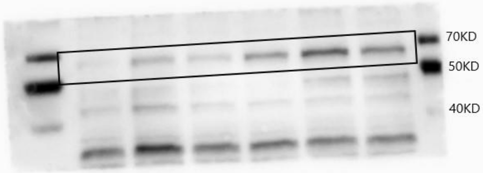

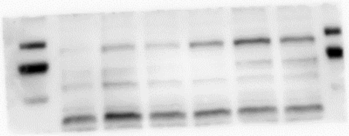

PP65

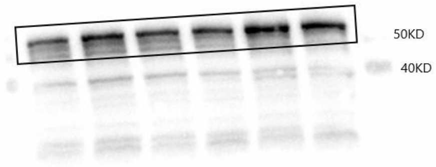

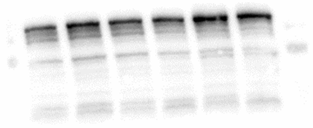

Supplement: Supplementary file 1 — Supplementary Information [file 41598_2021_85145_MOESM1_ESM.pdf]
